# Supplementary figures and images for: Slow manifolds within network dynamics encode working memory efficiently and robustly
Source: PLoS Comput Biol. 2021 Sep 15;17(9):e1009366. doi: 10.1371/journal.pcbi.1009366 (PMC8475983; doi:10.1371/journal.pcbi.1009366)

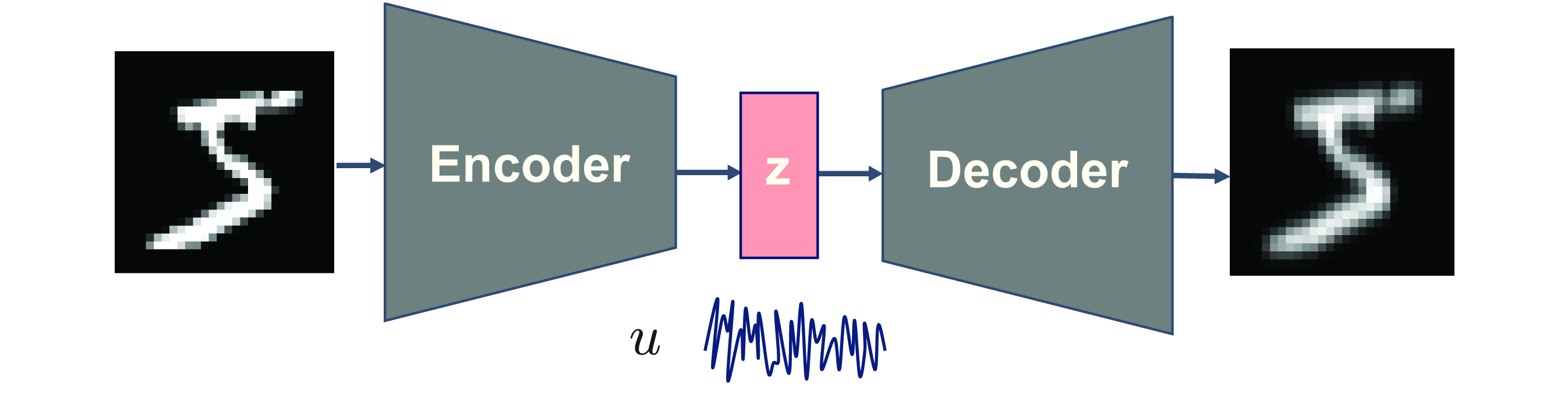

Supplement: S1 Fig — Several concatenated trials of the task with outputs zd=(zd1,zd2) and zo are shown. Using the modified FORCE method the network generates memory encodings during Td and trial output during response intervals, Tr (shaded area). (TIF) [file pcbi.1009366.s001.tif]

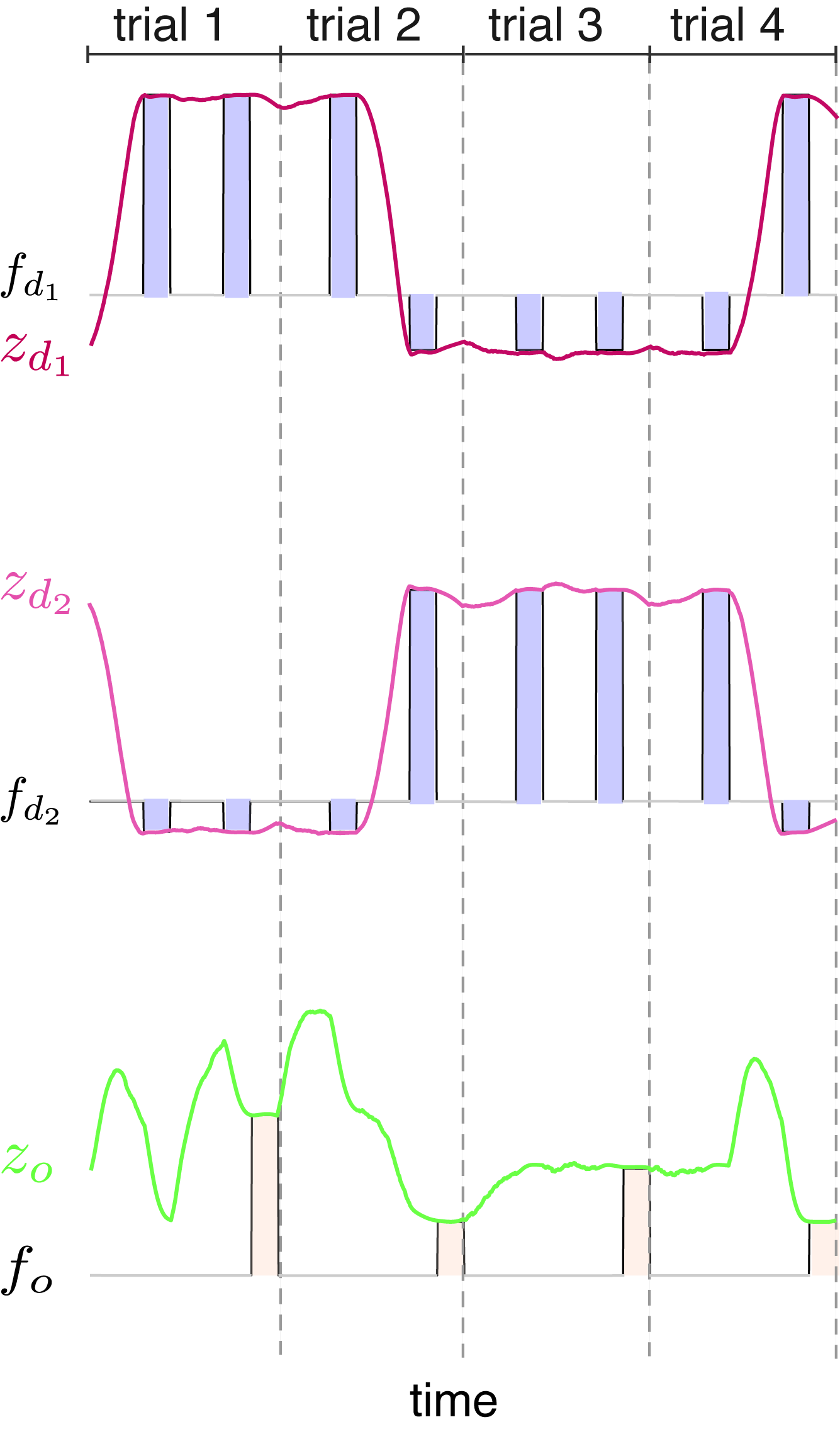

Supplement: S2 Fig — Categorization of dynamical mechanisms based on (i) the type of asymptotic attractor (either fixed point or limit cycle) and (ii) whether delay periods correspond to a fixed point. (TIF) [file pcbi.1009366.s002.tif]

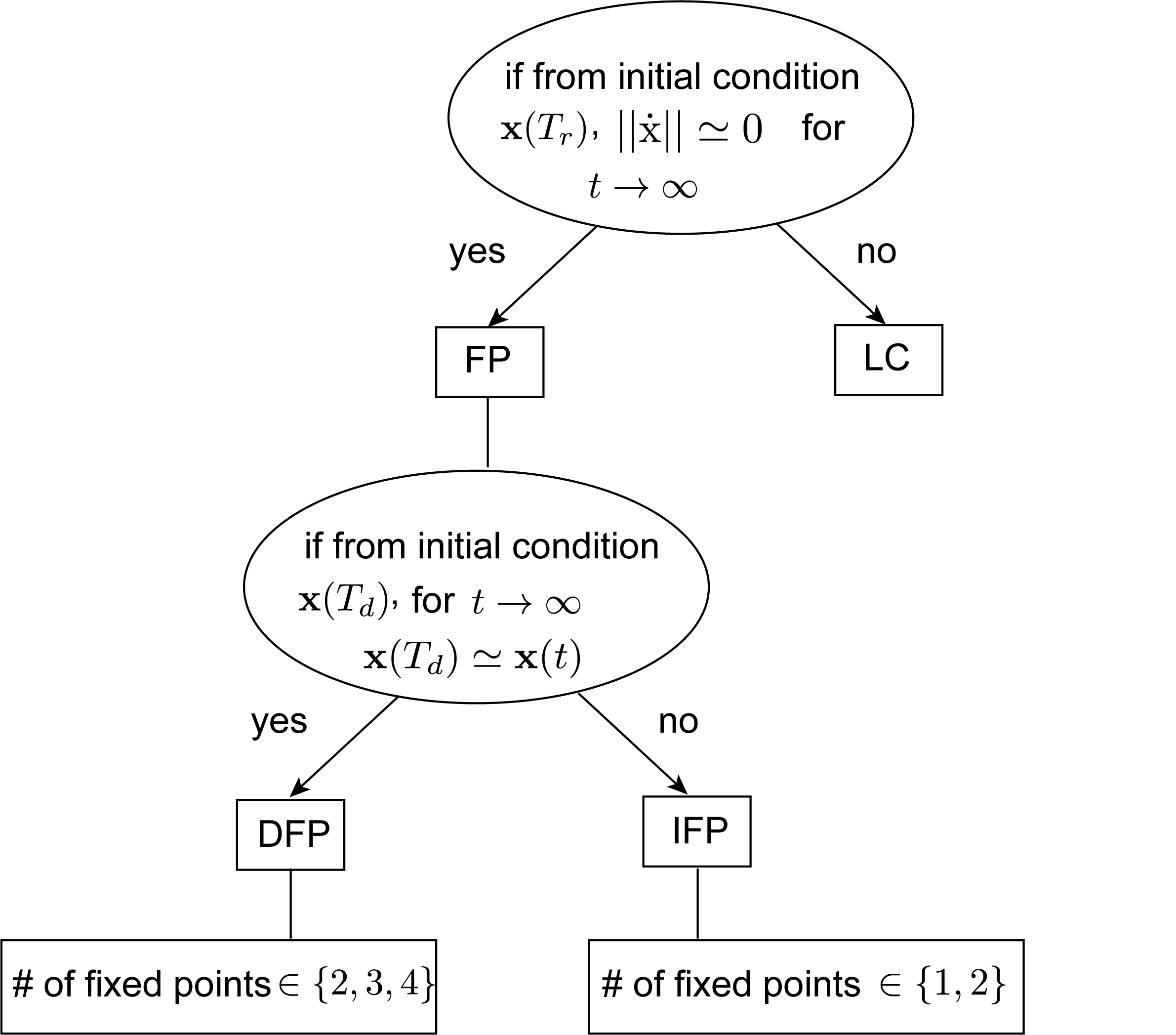

Supplement: S3 Fig — For the same exemplar networks as in Fig 3c, but a different set of stimuli (i.e., here, two realizations of the same digit are presented), neural activity and associated low-dimensional (PCA) trajectories are plotted. Note that PCA components are obtained for each exemplar network individually. The trajectories are color coded using the same scheme as the color bar on the top. In DFP, the network creates four stable fixed points to solve the SPM task (the inset shows the area inside the circle). For the displayed trajectory, the network uses two fixed points (shown in yellow) to represent memory and trial output. In IFP, memory representation and trial output are encoded along the slow manifold of the single fixed point in the state space. In LC, the trajectories approach a stable limit cycle. For the mixed mechanism, both a stable fixed point and limit cycle can be seen. (TIF) [file pcbi.1009366.s003.tif]

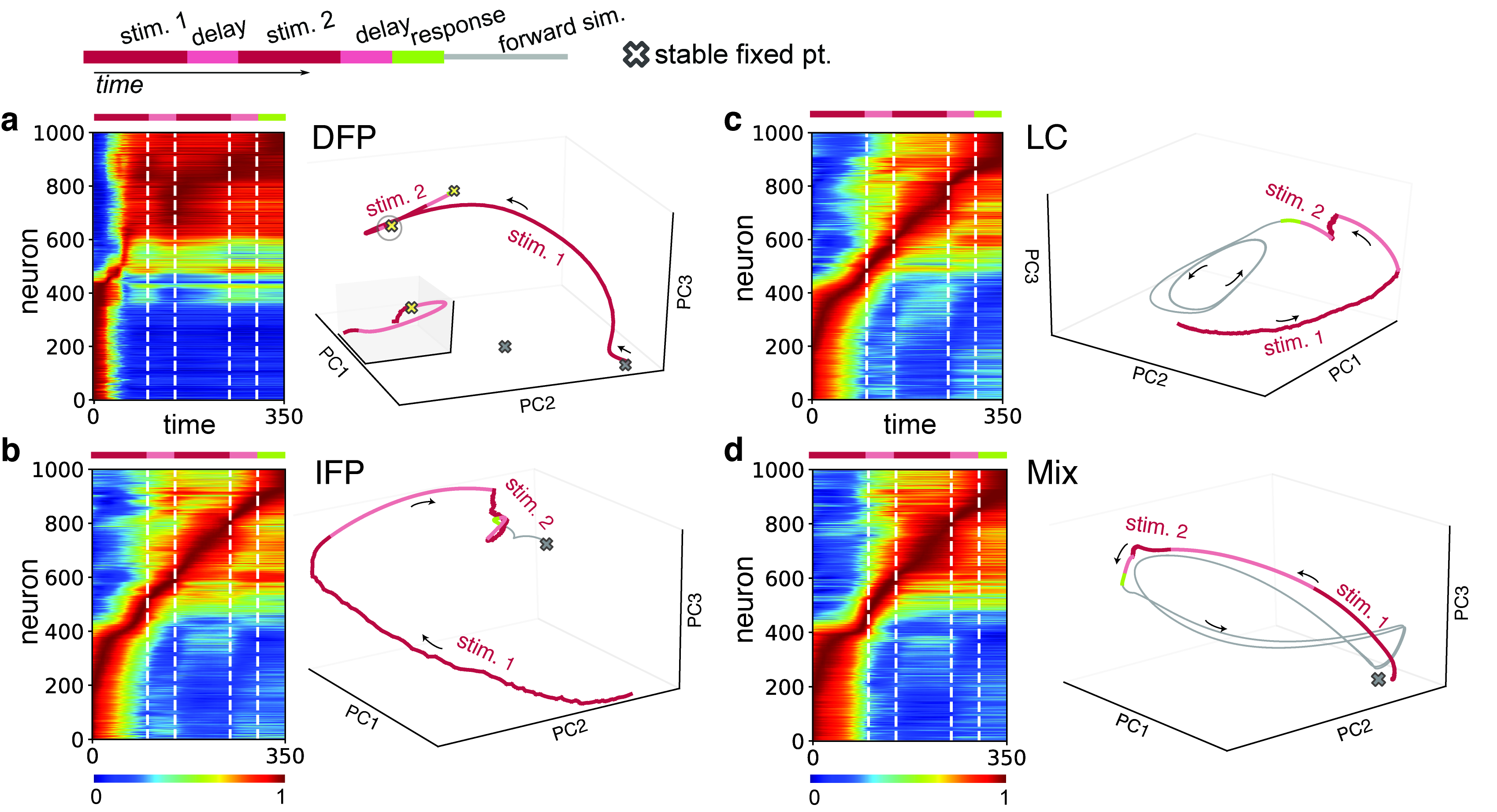

Supplement: S4 Fig — Plot shows how noise corrupts the salient trajectory for all four mechanisms (same trial and initial condition). PCs are exclusive to each network. In DFP, distracting noise places the trajectory in an erroneous basin of attraction and thus the network generates an incorrect response; in IFP noise pushes the trajectory away from the ‘correct’ slow manifold. (TIF) [file pcbi.1009366.s004.tif]

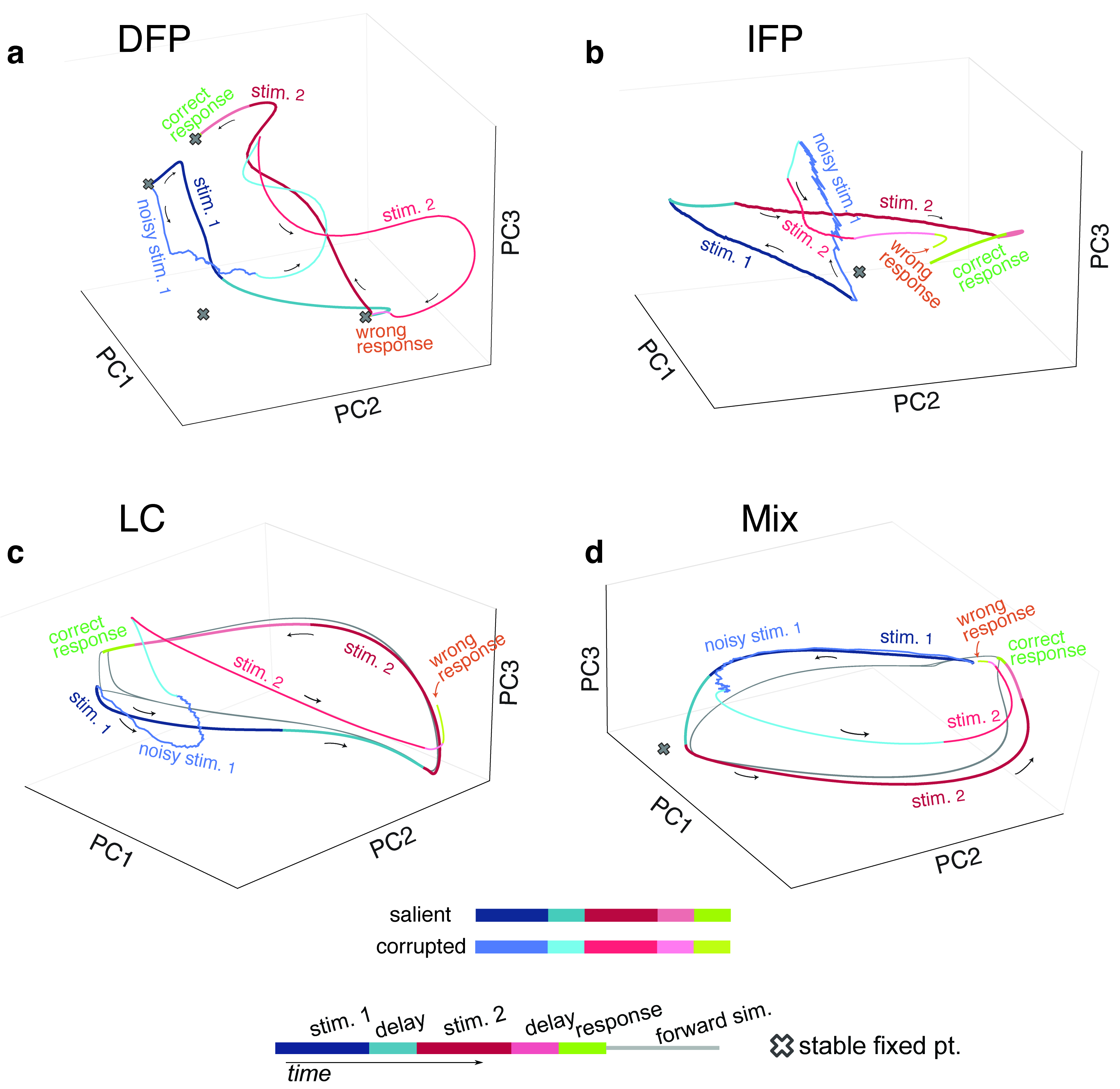

Supplement: S5 Fig — The latent space of VAE (i.e. z) is used to construct the input to the RNN model, where we have chosen a 2 dimensional latent space to represent x and y coordinates of MNIST digits dataset. For example, for x coordinate we obtain μx and σx corresponding to each specific digit and we can generate samples of that digit representation as temporal inputs (i.e. Gaussian process) to the network. (TIF) [file pcbi.1009366.s005.tif]
